# Impaired fear recognition and attentional set-shifting is associated with brain structural changes in alcoholic patients

**DOI:** 10.1111/adb.12175

**Published:** 2014-08-06

**Authors:** Leanne Trick, Matthew J Kempton, Steven C R Williams, Theodora Duka

**Affiliations:** 1 School of Psychology, University of SussexUK; 2 Department of Neuroimaging, Institute of Psychiatry, King's College LondonUK

**Keywords:** Anger, disgust, facial expression recognition, IED shift and reversal, inferior frontal gyrus, insula, VBM

## Abstract

Alcoholic patients with multiple detoxifications/relapses show cognitive and emotional deficits. We performed structural magnetic resonance imaging and examined performance on a cognitive flexibility task (intra-extradimensional set shift and reversal; IED). We also presented subjects with fearful, disgust and anger facial emotional expressions. Participants were abstaining, multiply detoxified (MDTx; *n *= 12) or singly detoxified patients (SDTx; *n *= 17) and social drinker controls (*n *= 31). Alcoholic patients were less able than controls to change their behavior in accordance with the changing of the rules in the IED and they were less accurate in recognizing fearful expressions in particular. They also showed lower gray matter volume compared with controls in frontal brain areas, including inferior frontal cortex (IFC) and insula that mediate emotional processing, inferior parietal lobule and medial frontal cortex that mediate attentional and motor planning processes, respectively. Impairments in performance and some of the regional decreases in gray matter were greater in MDTx. Gray matter volume in IFC in patients was negatively correlated with the number of detoxifications, whereas inferior parietal lobule was negatively correlated with the control over drinking score (impaired control over drinking questionnaire). Performance in IED was also negatively correlated with gray matter volume in IFC/BA47, whereas recognition of fearful faces was positively correlated with the IFC gray matter. Repeated episodes of detoxification from alcohol, related to severity of dependency, are coupled with altered brain structure in areas of emotional regulation, attention and motor planning. Such changes may confer increased inability to switch behavior according to environmental demands and social incompetence, contributing to relapse.

## Introduction

Chronic excessive use of alcohol can lead to impairments in many aspects of cognitive performance [e.g. visuospatial and verbal/non-verbal memory (Sullivan *et al.* 2002)] and has been the subject of recent meta-analysis (Stavro, Pelletier & Potvin 2012) showing impairments across 12 domains of cognitive function. Previous research has especially highlighted effects on tasks sensitive to prefrontal function; for instance Dirksen *et al.* (2006) found impairments in chronic alcoholics on the Wisconsin card sorting task (WCST) and Kamarajan *et al.* (2004) showed deficits on a Go-No go task. Although it is not clear what factors associated with alcohol drinking are responsible for these deficits, drinking history has been shown to relate to cognitive deficits associated with prefrontal function (e.g. Tarter & Ryan 1983) and cognitive functions have been shown to recover during abstinence (e.g. Sullivan *et al.* 2000) excluding the possibility that these cognitive deficits are pre-morbid.

In addition to cognitive changes, alcoholism has been associated with brain damage. Imaging methods have revealed enlarged ventricles in alcoholics compared with controls (e.g. Wobrock *et al.* 2009) and volume reductions in alcoholics in several brain areas including hippocampus (e.g. Nagel *et al.* 2005) and frontal lobes (for a review, see Buhler & Mann 2011).

Increasingly, the relationship between cognitive performance and structural brain changes in alcoholism is being examined. With a particular focus on executive function, Jang *et al.* (2010) investigated associations between brain structural changes and neuropsychological performance in alcoholics. In patients, perseverative responses and perseverative errors in the WCST correlated negatively with superior temporal gyrus and postcentral gyrus volumes, while WCST total errors correlated negatively with postcentral gyrus volume. Chanraud *et al.* (2007) also focused on executive function in relation to brain changes observed in alcoholics. They found that gray matter decreases in cerebellum and letter-number sequencing performance were correlated; volumes in superior frontal, precentral, postcentral (frontal and temporal cortices), insula and hippocampus were correlated with trailmaking performance; volumes in middle temporal gyrus, parahippocampus, thalamus and cerebellum were correlated with WCST perseveration errors. Fein *et al.* (2009) showed that parietal gray matter loss is related to spatial processing deficits. These studies all strongly suggest that brain structural changes related to alcoholism are associated with specific impairments in cognitive performance seen in this patient group. Such deficits, as for instance lack of cognitive flexibility (i.e. inability to switch away from inappropriate behavior as measured by the WCST), can increase the risk for relapse in abstinent alcoholics.

Alcohol-dependent populations also show deficits in another important function that may contribute to increased risk of relapse. Several studies have shown alcoholic patients being unable to recognize facial emotional expression in others (e.g. Townshend & Duka 2003). The ability to infer others' emotions is essential to successful social interactions and disruption could compound the interpersonal problems, such as lack of emotional support and social isolation, often experienced by alcoholic patients (Maurage *et al.* 2011).

In recent years, research in animals and humans has pointed to the role of repeated withdrawal from alcohol in brain damage and cognitive and emotional impairments (Stephens & Duka 2008; Duka *et al.* 2011). For instance repeated exposure to alcohol intoxication and subsequent withdrawal in animals is not only associated with cognitive deficits but also with brain damage (Obernier, Bouldin & Crews 2002; Stephens *et al.* 2005). Duka *et al.* (2003) confirmed in an alcoholic patient population that cognitive deficits increased with the number of previous detoxifications—patients with experience of multiple detoxifications made more errors on the Wechsler Adult Intelligence Scale (WAIS) maze task and the vigilance task from the Gordon diagnostic battery and were less able to withhold a response to receive a reward than patients who had experienced only a single detoxification. Loeber *et al.* (2010) also found that alcoholic patients with most previous detoxifications were less able to learn to choose cards from the more advantageous decks compared with controls in the Iowa gambling task. Thus, although other factors associated with alcohol dependence may contribute to cognitive impairments seen in alcoholic patients (e.g. Sullivan *et al.* 2002), repeated withdrawal from alcohol seems to be an important factor in its own right. On the basis of evidence from animal experiments, it has been suggested that repeated withdrawal from alcohol results in an adaptation of brain mechanisms, similar to that occurring during epileptic kindling (Stephens *et al.* 2005; Stephens & Duka 2008).

The present study aims to extend previous work, which has shown impairments in cognitive functions in alcoholic patients, to examine the deficits in cognitive functions associated with risk of relapse (i.e. flexible behavior and social cognition) and to show brain structural changes found in alcoholics, which are associated with these deficits. In particular, the present study aims to demonstrate that the number of prior detoxifications will be related linearly to both brain structural changes and performance impairments.

Previous studies of cognitive function in alcoholism have used the WCST (e.g. Oscar-Berman *et al.* 2009), which has been proposed as the ‘gold standard’ measure of executive function (Royall, Chiodo & Polk 2003). In the present study, the intra-extradimensional set shift (IED) was used, a computerized analogue of the WCST. The IED task is expected to measure the ability to switch behavior when not appropriate; perseveration in a response which is no longer rewarded is the major performance outcome. Scaife & Duka (2009) found impairments in IED performance in binge drinkers compared with non-binge drinkers. It has been proposed that the pattern of repeated episodes of heavy drinking combined with withdrawal observed in alcoholics can also be seen in a milder form in binge drinking, thus the IED task is expected to be sensitive to deficits in alcoholics with the most experiences of withdrawal. Social cognition was measured by the emotional facial recognition task. In this task, alcoholic patients are shown to be impaired especially in recognition of fear, anger and disgust (e.g. Townshend & Duka 2003).

## Method

### Study population

Sixty participants were included in the analysis. Twenty-nine alcohol-dependent patients were recruited from diagnosed alcoholics seeking treatment as in-patients (Bethlem Royal Hospital, Beckenham) or outpatients (Crosfield House, Croydon) at the South London and Maudsley NHS Foundation Trust, UK. A control group of mild to moderate social alcohol drinkers were recruited from the local community in central London and Croydon via announcements on information boards and in local newspapers (control group; *n *= 31). The control group had no current or previous alcohol-related problems as defined by DSM-IV.

The patient population was subsequently divided into two groups regarding medically supervised detoxifications (MSDs; events clearly described in medical records as periods of abstinence under medical supervision). The two groups consisted of (1) those patients with two or fewer (including current) MSDs (single detoxification group; SDTx, *n *= 17); and (2) those patients with more than two MSDs (multiple detoxification group; MDTx, *n *= 12). The use of the criterion > 2 detoxifications to classify the MDTx group was based on previous studies (Duka *et al.* 2002; O'Daly *et al.* 2012).

Participants were aged 25 to 65 years, weighed 50 to 90 kg and were in generally good physical condition, as documented by their medical history. All participants were right-handed and able to speak and write English. Alcohol dependence was diagnosed by independent clinicians according to the DSM-IV (American Psychiatric Association 1994) or ICD-10 (WHO 1993). All patients had been abstinent for a minimum of 2 weeks at the time of the study and had been medically supported during withdrawal with standard detoxification treatments, including administration of chlordiazepoxide and thiamine. All patients had ceased benzodiazepine treatment at least 72 hours prior to testing. Volunteers who constituted the control group of social drinkers were not included if their medical history suggested psychiatric, neurological or other chronic disorders, or if they were currently undergoing any drug treatment interfering with the scope of the trial. Participants also had to be suitable for a magnetic resonance imaging (MRI) scan and during the testing session, smoking and caffeine or other foods containing xanthine derivatives (e.g. coffee, tea, chocolate or cola drinks) were not permitted. At the recruitment stage, volunteers were given detailed oral and written information inviting them to take part and explaining the procedures and restrictions. Before commencement of the testing session, volunteers were given an information sheet and asked to give their written consent for participation. The study was approved by the Kings College Hospital NHS Research Ethics Committee.

### Design and procedure

The experiment lasted approximately 2.5 hours for each participant (the tasks took approximately 30 minutes to complete). Other data including functional MRI obtained during the same scanning session are presented elsewhere (O'Daly *et al.* 2012).

### Materials

#### Questionnaires

Several questionnaires were given to the participants. Some of the questionnaire data have been previously presented (O'Daly *et al.* 2012). Here the impaired control over drinking questionnaire (ICQ) (Stockwell *et al.* 1994), severity of alcohol dependence questionnaire (SADQ) (Stockwell, Murphy & Hodgson 1983) and the state trait anxiety inventory are presented again. Demographic data including verbal intelligence quotient (IQ) are also presented.

#### IED: CANTAB (Cambridge Cognition Ltd., Cambridge, UK)

IED is a test of rule acquisition and reversal requiring visual discrimination, attentional set formation and maintenance, shifting and flexibility of attention. It is primarily sensitive to changes in the frontostriatal areas of the brain. Two artificial dimensions are used in the test: color-filled shapes and white lines. Simple stimuli are made up of just one of these dimensions, whereas compound stimuli are made up of both, namely white lines overlying color-filled shapes. First, participants see two simple color-filled shapes and must learn which one is correct by touching it. Feedback indicates which stimulus is correct, and after six correct responses, the stimuli and/or rules are changed. These shifts are initially intra-dimensional (e.g. color-filled shapes remain the only relevant dimension), then later extra-dimensional (white lines become the only relevant dimension). Participants progress through the test by satisfying a set criterion of learning at each stage (six consecutive correct responses). If at any stage the participant fails to reach this criterion after 50 trials, the test terminates. The task consists of nine stages including single and compound discrimination (adjacent and overlapping) and reversal, as well as intra-dimensional and extra-dimensional shift and reversal.

IED outcome measures are based on errors and number of trials, with both greater errors and more trials indicating poorer performance. Completed stage trials and errors comprised the dependent variables and were analyzed using one-way ANOVA with group (control, SDTx, MDTx) as the between subjects factor. Significant main effects were interpreted using appropriate pairwise comparisons.

#### Facial emotion recognition task

The task was programmed using E-Prime v1.1 (Psychology Software Tools, Philadelphia, PA, USA) and presented on a Dell X1 laptop computer. Visual stimuli were black and white faces displayed on a grey background (Townshend & Duka 2003). Text font and size was Times New Roman 20pt. The task comprised 50 trials. In each trial, one face was presented individually in the center of the screen. The faces displayed either a neutral emotion (10 faces), a fearful emotion (10 faces), a morph of 50 percent neutral and 50 percent fearful emotion (10 faces), a disgust emotion (10 faces) or an angry emotion (10 faces). Half of the faces were male and half were female. Presented underneath the faces in each trial were the instructions ‘Press a number key between 1 and 7 to indicate what emotion you see in this face. 1 = HAPPINESS, 2 = SURPRISE, 3 = FEAR, 4 = SADNESS, 5 = DISGUST, 6 = ANGER, 7 = NEUTRAL’. Outcome measures were percentage correct responses for each emotion (correct response for the 50 percent neutral and 50 percent fear morph was fear).

All analyses were performed using SPSS 16.0 (SPSS Inc., Chicago, IL, USA).

### MRI methods and analysis

#### Acquisition of MRI scans

Anatomical images were acquired using a 3T General Electric Signa System MRI scanner at the Maudsley Hospital, London. Images were acquired in the coronal plane using a T_1_-weighted, three-dimensional spoiled gradient recalled echo protocol (echo time = 2.8 milliseconds, repetition time = 7.0 milliseconds, inversion time = 450 milliseconds, flip angle = 20°, slice thickness = 1.1 mm, in plane resolutio*n *= 1.09 × 1.09 mm, number of excitations = 1).

#### Analysis

All images were checked manually for gross structural abnormalities before analysis and flipped into the axial plane. Analysis was performed using voxel-based morphometry (VBM) with unified segmentation in SPM5 (www.fil.ion.ucl.ac.uk/spm/software/spm5) (Ashburner & Friston 2005). This version of VBM was developed after optimized VBM but before the DARTEL (Diffeomorphic Anatomical Registration Through Exponentiated Lie Algebra) processing method was introduced. Unified segmentation performs image registration, MRI bias field correction and tissue segmentation in one generative model; although more complex, this integrative method is likely to be more accurate than sequential processing steps such as ‘optimized VBM’ (Good *et al.* 2001). We used the standard segmentation option in SPM5 with Cleanup partitions set to ‘Thorough Clean’ to ensure non-brain tissue was excluded from the gray matter segmentations. Normalized and modulated gray matter segmented images were produced for each subject. Images were smoothed using a Gaussian isotropic kernel of 12 mm full width half maximum.

To calculate total intracranial volume (TIV), modulated white matter and cerebrospinal fluid (CSF) images were also produced for each subject, and intracranial volume was calculated by summing the total gray matter, white matter and CSF fractions. There was no significant difference in TIV between the groups [*F*(2,57) = 1.016, *P *= 0.368]. However, as regional brain volume typically correlate with TIV, we included TIV as a covariate to remove additional variance. In addition, as gray matter volume negatively correlates with age, age was also entered as a covariate in the analysis.

We used a voxel-based whole-brain analysis to ensure that we captured all regional group differences. We used an analysis of covariance (ANCOVA) model in SPM5 with TIV and age as covariates to examine the effect of group (controls, SDTx, MDTx) on regional gray matter volume. Suprathreshold clusters were identified using a threshold of *P* < 0.05 [family-wise error (FWE) corrected] and cluster extend threshold k > 0, and MNI coordinates produced by SPM5 were converted to Talairach coordinates (Duncan *et al.* 2000). In regions where there were significant group differences, we used MarsBaR (Brett *et al.* 2002) to extract gray matter volumes from clusters in SPM5 from each subject; these were subsequently used in ANCOVA models implemented in SPSS 16.0, to investigate potential differences between controls, SDTx patients and MDTx patients. TIV and age were used as covariates and *post hoc* pairwise comparisons used Bonferonni correction.

### Correlations

Second-order partial correlations controlling for the effects of TIV and age were performed to investigate relationships between gray matter volume in brain regions where there was a main effect of group and (1) alcohol dependence measurements including number of detoxifications, SADQ and ICQ scores; (2) IED dependent variables (number of trials and errors of completed stages); and (3) percentage correct responses in the facial emotion recognition task for 100 percent fear, anger and disgust morphs. Correlations significant at the *P* < 0.05 level are reported. Corrections for multiple comparisons for correlations were not applied allowing for the r values as effect size (Field 2013) to support interpretation of the findings.

## Results

### Demographic and clinical characteristics

The groups were well matched in terms of age, gender and full scale IQ. Population characteristics are given in Table 1. While controls and patients differed in terms of self-reported daily alcohol units as expected, the two patient groups, SDTx and MDTx, were matched for this variable. One individual in each group of patients was an outlier (see Table 1, range). Exclusion of these individuals from the analysis did not change significance levels.

**Table 1:** Population characteristics for controls and alcoholic patients with one (SDTx) or more (MDTx) previous detoxifications. Values given in mean (standard deviation) except for units drunk per day for which values reported are median (and range)

Variable	Controls (*n =* 31)	SDTx (*n =* 17)	MDTx (*n =* 12)	Statistical analysis
Age (years)	40.2 (8.7)	37.6 (9.6)	44.4 (9.5)	*F*(2,59) = 1.79, n.s.
Gender	16M, 15F	11M, 6F	7M, 5F	χ^2^(2) = 0.78, *P *= 0.676
Full scale IQ (from NART)	106.7 (7.3)	101.9 (7.1)	106.3 (6.3)	*F*(2,59) = 2.62, n.s.
Supervised detoxifications^a^	N/A	1.3 ± 0.1	4.3 ± 0.4	t(12.6) = −6.65, *P* < 0.001
Cigarettes/day	10.8 (4.0)	24.6 (18.1)	23.6 (8.2)	*F*(2,27) = 1.192, *P *= 0.169
Alcohol units^b^ drunk per day	1.8 (0–9)	28.0 (10–187.90)	39.7 (20–150)	χ^2^(2) = 44.9, *P* < 0.001

a Significant differences between SDTx and MDTx groups.

b One unit = 8 g of alcohol (UK defined unit of alcohol). F = female; IQ = intelligence quotient; M = male; MDTx = multiple detoxification group; NART = National Adult Reading Test; N/A = not applicable; n.s. = not significant; SDTx = single detoxification group.

The ICQ and SADQ questionnaires were completed by all alcohol-dependent participants. The two patient groups did not differ on the ICQ [t(27) = −1.56, *P *= 0.090], but MDTx had higher total SADQ compared with SDTx [t(23.7) = −3.48, *P *= 0.002].

ICQ, SADQ as well as anxiety data are presented in Table 2.

**Table 2:** Impaired control over drinking (ICQ) and severity of alcohol dependence (SADQ total) as well as trait and state anxiety. Values given in mean (standard deviation)

Variable	Controls (*n =* 31)	SDTx (*n =* 17)	MDTx (*n =* 12)	Statistical analysis
ICQ	N/A	11.2 (2.6)	12.7 (1.4)	t(27) = −1.56, *P *= 0.090
Total SADQ	N/A	33.1 (14.3)	46.8 (6.4)	t(23.7) = −3.48, *P *= 0.002
State anxiety (STAI)^a^	30.2 (7.6)	38.8 (9.9)	39.7 (14.2)	*F*(2,59) = 6.35, *P* < 0.01
Trait anxiety (STAI)^a^	36.6 (9.8)	50.6 (10.3)	46.3 (12.1)	*F*(2,59) = 10.82, *P* < 0.001

a Significant difference between controls and patients (*Ps* < 0.01), but not SDTx and MDTx *P* > 0.268. MDTx = multiple detoxification group; N/A = not applicable; SDTx = single detoxification group; STAI = state trait anxiety inventory.

Participants were recreational drug users among patients and controls. There was no history of drug use disorder present in any of the participants. Recreational drug users, were *n *= 12 in control group, *n *= 9 in SDTx and *n *= 8 in MDTx group. There was no difference between groups with regard to number of cigarettes smoked per day (Table 1). However, there were more smokers among the patient groups (*n *= 9 in MDTx and *n *= 14 in SDTx) than among the controls (*n *= 8).

### IED

**Figure 1:**
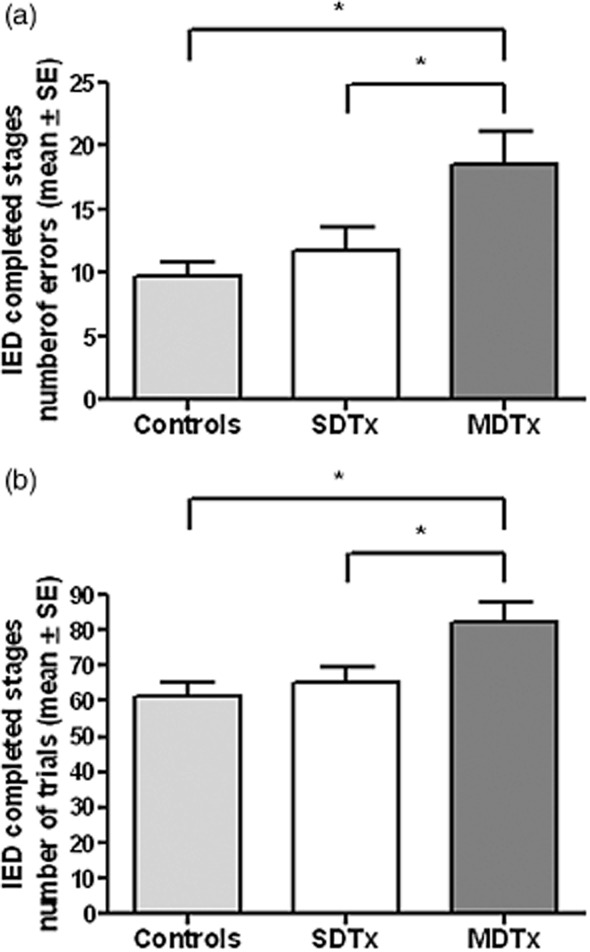
Number of errors (a) and trials (b) of completed stages (mean ± SEM) in controls and alcoholic patients with one (SDTx) or more (MDTx) previous detoxifications; *P < 0.05. Between-group differences were retained using non-parametric tests (Kruskal–Wallis one-way ANOVA test for the three group comparison and Mann–Whitney U-test for comparing MDTx and SDTx groups)

IED data were available for a total of 57 participants (30 controls, 16 SDTx, 11 MDTx). For all completed stages combined, MDTx patients made more errors and took more trials to complete each stage compared with controls (*P* < 0.01) and SDTx patients [*P* < 0.05; main effect of group: *F*(2,54) = 6.59, *P *= 0.003 for errors and *F*(2,54) = 5.19, *P *= 0.009 for trials] (see Fig. 1).

### Facial emotion recognition task

**Figure 2:**
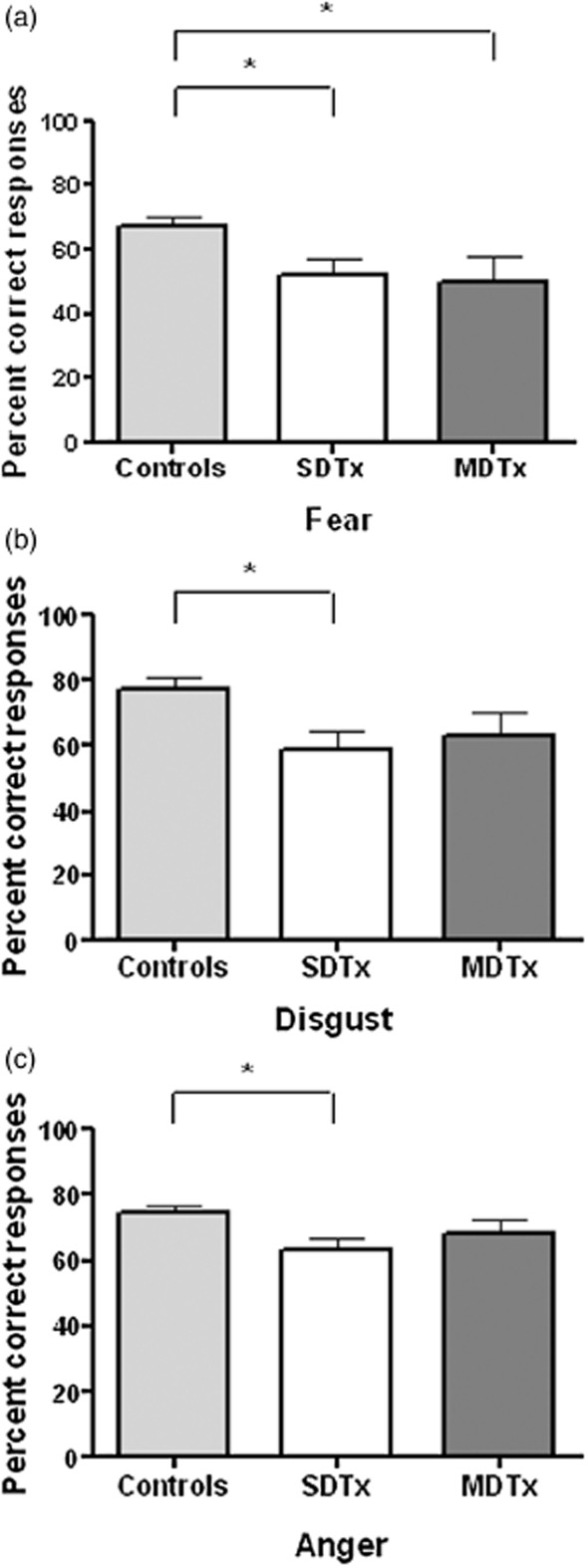
Percent correct responses (mean ± SEM) for fear (100 percent morph, a), disgust (b) and anger (c) recognition in controls and alcoholic patients with one (SDTx) or more (MDTx) previous detoxifications; *P < 0.05. Between-group differences were retained using non-parametric tests [Kruskal–Wallis one-way ANOVA test for the three group comparison, except for anger recognition (*P *= 0.053)]

Data were available for 59 participants (30 controls, 17 SDTx, 12 MDTx). Percentage correct responses were lower for both SDTx patients (*P *= 0.027) and MDTx patients (*P *= 0.023) compared with controls in identifying fearful faces [100 percent fear; *F*(2,56) = 3.59, *P *= 0.034]. Percentage correct responses to disgust faces [*F*(2,56) = 4.97, *P *= 0.010] and anger faces [*F*(2,56) = 4.02, *P *= 0.023] were also lower in SDTx patients compared with controls (*P *= 0.005 and *P *= 0.011, respectively). There were no significant group differences in percent correct responses to the 50 percent neutral and 50 percent fear morph, or to the neutral face. Figure 2 depicts the findings.

### Structural MRI

#### Global gray matter, white matter, CSF and TIV

Control participants had greater gray matter volume than SDTx patients (*P *= 0.014) and MDTx patients (*P *= 0.001). Control participants also had greater white matter volume than SDTx patients (*P *= 0.009) and MDTx patients (*P *= 0.026). Finally, control participants had smaller CSF volume than SDTx patients (*P *= 0.001) and MDTx patients (*P* < 0.001; see also Table 3 where main effects are given).

**Table 3:** Global gray matter, white matter, CSF and TIV volumes (liters) by group. Values given in mean (standard deviation)

Variable	Controls (*n =* 31)	SDTx (*n =* 17)	MDTx (*n =* 12)	ANCOVA
Gray matter[Table-fn tf3-1]	0.682 (0.08)	0.667 (0.04)	0.624 (0.06)	*F*(2,56) = 7.09, *P *= 0.002
White matter^a^	0.474 (0.05)	0.454 (0.05)	0.438 (0.06)	*F*(2,56) = 4.82, *P *= 0.012
CSF^a^	0.457 (0.10)	0.550 (0.13)	0.523 (0.08)	*F*(2,56) = 9.83, *P* < 0.001
TIV	1.613 (0.19)	1.671 (0.15)	1.584 (0.15)	*F*(2,57) = 1.02, *P *= 0.368

a Controls significantly different from SDTx and MDTx, pairwise statistics given in text. CSF = cerebrospinal fluid; MDTx = multiple detoxification group; SDTx = single detoxification group; TIV = total intracranial volume.

#### Voxel-based whole-brain analysis

**Figure 3:**
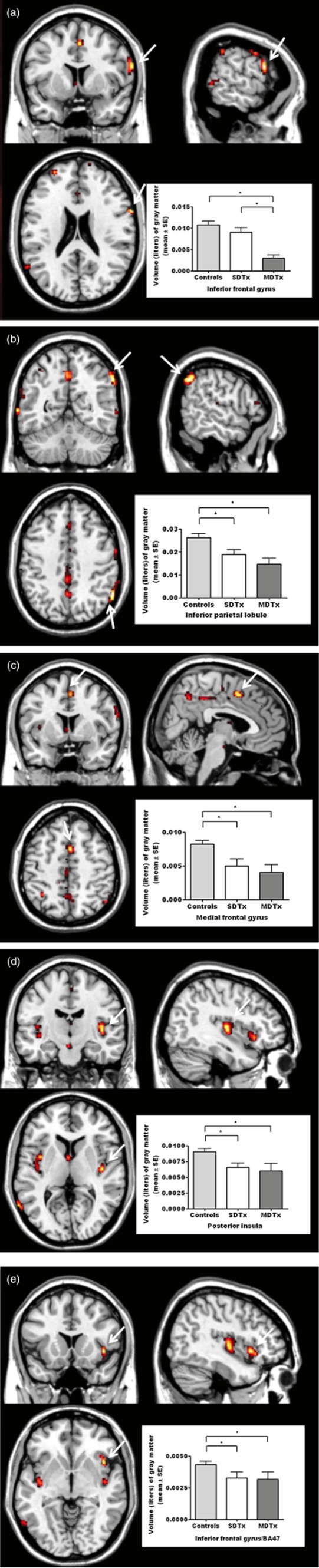
Regions showing differences of gray matter volume between the three groups (main effect of group: controls > SDTx > MDTx); (a) inferior frontal gyrus; (b) inferior parietal lobule; (c) medial frontal gyrus; (d) posterior insula; (e) inferior frontal gyrus (BA47). Clusters are shown using a height threshold of *P* < 0.0001 uncorrected, for illustrative purposes

A main effect of group on gray matter volume (*P* < 0.05 FWE corrected) was identified in several right prefrontal cortical areas including the inferior frontal gyrus (IFG), inferior parietal lobule/BA40, medial frontal gyrus/BA32, posterior and a cluster of inferior frontal cortex (BA47) neighboring anterior insular (see Table 4). With the exception of the IFG area, MDTx and SDTx patients showed in all other identified areas reduced gray matter volume compared with controls (*P*s < 0.05); in the IFG area, MDTx patients showed reduced gray matter compared with both controls and SDTx patients (*P*s < 0.05; see Fig. 3).

**Table 4:** Voxel-based morphometry summary providing the areas where a significant main group effect was found

Region label [right (R)/left (L)]	Talairach coordinates	z score	Cluster size	*P* value (FWE)
Inferior frontal gyrus (R)	64, 8, 22	5.02	4	0.025
Inferior parietal lobule/BA40 (R)	57, −52, 41	4.99	8	0.028
Medial frontal gyrus/BA32 (R)	4, 8, 46	4.99	2	0.029
Posterior insula (R)	40, −13, 6	4.92	2	0.039
Inferior frontal gyrus/BA47 (R)	42, 15, −4	4.87	1	0.048

FWE = family-wise error correction.

### Correlations

#### Number of detoxifications, ICQ and SADQ

**Figure 4:**
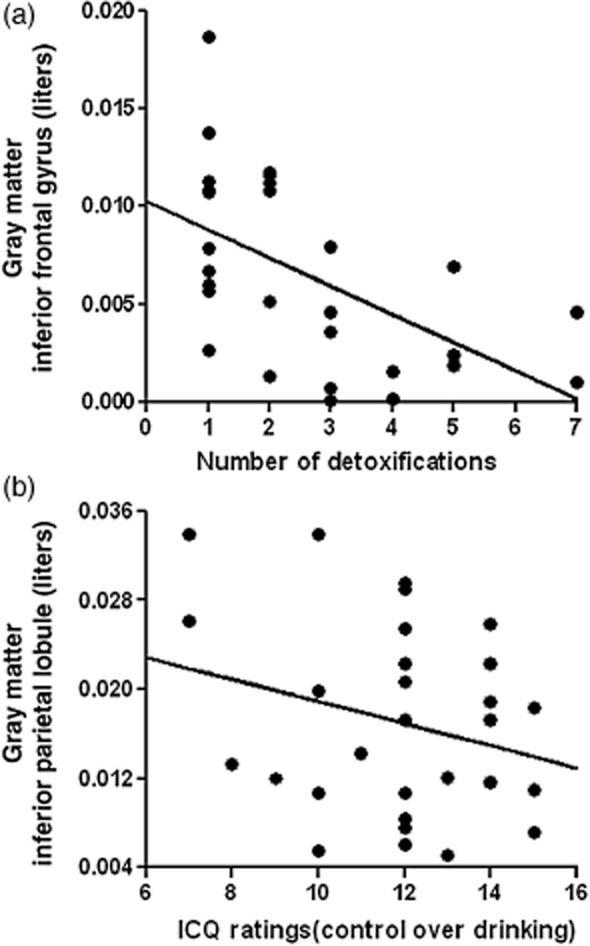
Correlations depict the negative relationships between gray matter volumes in inferior frontal gyrus (raw data) and number of detoxifications (a; r = −0.47, *P *= 0.017) as well as between gray matter volumes in inferior parietal lobule (raw data) and ratings of inability to control drinking (b; r = −0.42, *P *= 0.030) in the patient group

Significant negative correlations were found between gray matter volume in IFG and the number of medically supervised detoxifications (patients only; r = −0.47, *P *= 0.017, Fig. 4a), indicating that greater numbers of attempts to quit are related to lower gray matter volume in this area in alcohol-dependent patients. To examine a possible role of smoking habits to this relationship between number of detoxifications and gray matter volume in IFG, *post hoc* partial correlations were performed between gray matter volume in IFG and number of cigarettes smoked per day. No significant relationship was found (patients only; r = 0.011, *P *= 0.956).

Significant negative correlation were also found between gray matter volume in inferior parietal lobule and ICQ score (patients only; r = −0.42, *P *= 0.030, Fig. 4b), indicating a relationship between low gray matter volume in this area and inability to control drinking. No significant correlations were found with SADQ.

#### IED performance

**Figure 5:**
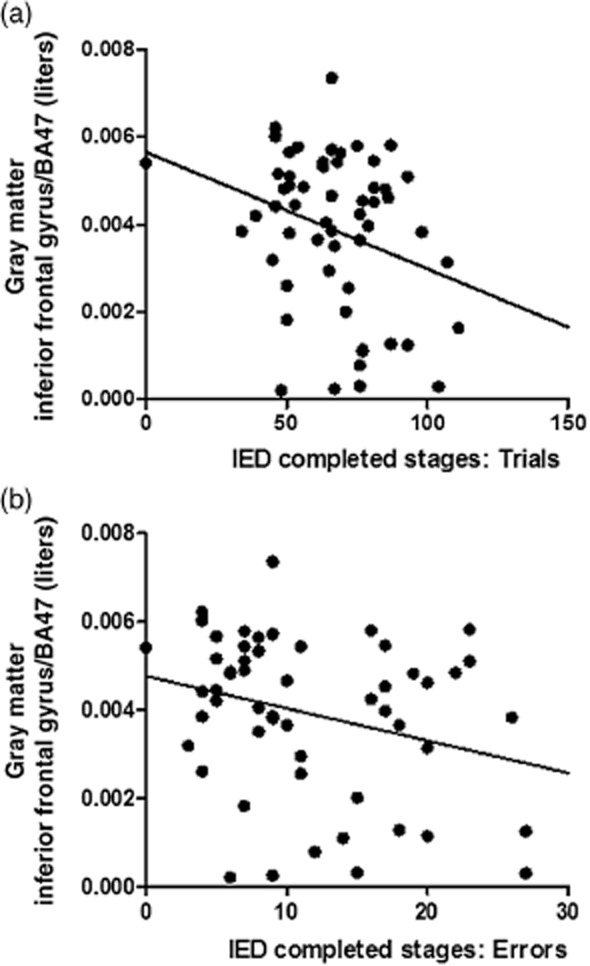
Correlations depict the negative relationships between gray matter volumes in inferior frontal gyrus (BA47; raw data) and IED number of trials (a; r = −0.272, *P *= 0.044) and errors (b; r = −0.291, *P *= 0.031) of completed stages

There were significant negative correlations between gray matter volume in IFG (BA47) and IED number of trials and errors of completed stages (r = −0.272, *P *= 0.044 and r = −0.291, *P *= 0.031, respectively; Fig.  5); and between gray matter volume in posterior insula and IED completed stages number of errors (r = −0.265, *P *= 0.05). In all cases, these correlations indicate that lower gray matter volumes are associated with impaired IED performance.

#### Facial emotion recognition

**Figure 6:**
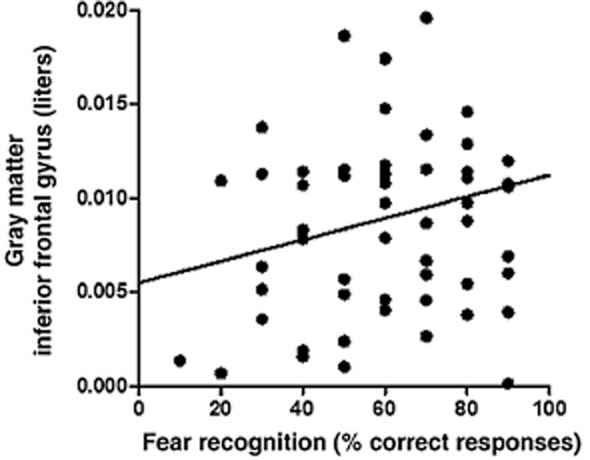
Correlation depicts the positive relationships between gray matter volumes in inferior frontal gyrus (raw data) and correct responses for fear (100 percent morph) recognition (r = 0.36, *P *= 0.006)

Correct responses of fear recognition were correlated with gray matter volumes in IFG (r = 0.36, *P *= 0.006; Fig. 6), whereas correct responses of disgust recognition were correlated with gray matter volumes in inferior parietal lobule (r = 0.33, *P *= 0.011), posterior insula (r = 0.34, *P *= 0.010) and medial frontal gyrus (r = 0.26, *P *= 0.048); correct responses for anger recognition were also correlated with gray matter volumes in medial frontal gyrus (r = 0.35, *P *= 0.007). In all cases, decreased volumes were related to poorer emotion recognition.

Although corrections for multiple comparisons for these correlations were not applied, the size effect of relationships (r values) between the important variables can still be used for interpretation of the findings.

## Discussion

The present study demonstrated that alcoholic patients are impaired in a cognitive flexibility task and in a social cognition task compared with controls. In addition, gray matter volume in several brain areas including IFG and parietal lobule as well as insula and medial frontal gyrus was found to be less compared with controls. Furthermore, as predicted, these changes were linearly related to detoxifications (controls > SDTx > MDTx) and the decrease in gray mater volume in the IFG as well as the impairment seen in performance in the attentional set-shifting task were significantly greater in MDTx compared with SDTx.

Brain areas found with reduced gray matter volume in alcoholic patients constitute areas involved in inhibitory control, attentional, motor as well as emotional processes, all important for regulation of appropriate cognitive and emotional responses. IFG is involved in attentional control and response inhibition (Hampshire *et al.* 2001) whereas medial frontal cortex supports selection of appropriate response (e.g. Rushworth, Kennerley & Walton 2005). Insula interprets the emotional input to regulate the emotional response and has been implicated in facial recognition of emotions (e.g. Phillips *et al.* 2004). Reduced gray matter in insula may be associated to the deficits seen in emotional facial recognition. Indeed, previous research has shown in alcohol-dependent participants decreased activation of insula when presented with fearful faces (O'Daly *et al.* 2012). Interestingly, the changes in activation seen in insula in the previous study (O'Daly *et al.* 2012) and in gray matter volume seen in the present study (in insula but also in the other brain areas) reflected a linear decrease across the three groups controls, SDTx and MDTx. Thus it seems that with increased experiences of detoxifications, brain structure and function becomes increasingly deficient. Furthermore, a direct negative relationship was found between the number of detoxifications that patients have gone through and the gray matter volume in IFG demonstrating that inhibitory control may be in particular sensitive to the number of quit attempts. This is consistent with Cardenas *et al.* (2011) who showed that early in abstinence brain volumes (particularly in regions of the mesocorticolimbic reward system) differentiate future relapsers from future abstainers, suggesting that volumetric changes are greatest in those alcohol-dependent patients who have undergone most detoxifications. In addition, it has been reported that lower processing speed seen in cognitive tasks including executive function tasks was associated with greater risk of relapse (Durazzo *et al.* 2008); this previous finding further supports the present findings of cognitive impairment in the attentional set-shifting task being greater in the MDTx group. However, it is unknown whether structural differences in this circuitry and differences in cognitive performance might confer greater risk for relapse, or if they are a consequence of multiple detoxifications. In addition to episodes of multiple detoxifications, other confounding factors (e.g. age of starting drinking heavily, length of alcohol use disorder) not examined here may also contribute to the deficits seen in the present cohort. In the present study, duration of abstinence was not obtained; thus it is not possible to provide data on how abstinence duration may relate to brain structure changes.

Interestingly, the two groups of patients differed in their SADQ ratings and SADQ was correlated with the number of detoxifications (r = 0.380, *P *= 0.042). Thus it seems that alcohol dependency measures are linked to number of detoxifications. This is not surprising as SADQ measures withdrawal symptoms (physical and affective withdrawal, withdrawal and craving relief drinking) known to be increased with the number of detoxifications (Ballenger & Post 1978). In addition, SADQ measures rapidity of reinstatement and alcohol consumption. Although number of units drunk per week was not different between the two patient's groups, values were based on self-reports, which cannot always be accurate.

Another important factor that may have contributed to brain structure changes is smoking. A limitation of the present study is that no detailed information was obtained with regard to smoking history of participants to explore this possibility. However, *post hoc* correlations between cigarettes smoked per day and gray matter volume (milliliters) in IFG showed no significant relationship.

Supporting the role of multiple detoxifications in the brain changes, correlations revealed that the number of detoxifications was negatively associated with gray matter volume in IFG, an area involved in inhibitory control. It is possible that decreased inhibitory control associated with damaged IFG supports the occurrence of repeated relapses. Inability to control drinking (ICQ ratings) on the other hand was associated with reduced gray matter in inferior parietal lobule, an area associated among other functions with selective attention (e.g. Mennemeier *et al.* 1994; Shomstein, Kravitz & Behrmann 2012), the recognition of affect (Mandal *et al.* 1999) and bodily sensory input (Cutting 1991). The latter function may contribute to maintenance of self awareness (Macuga & Frey 2011), an important function for successful control over drinking.

Performance in IED was negatively correlated with gray matter volume in a cluster within IFG (BA47). IFG has been implicated in previous research during cognitive set switching (Kim *et al.* 2011), a probabilistic response reversal (Budhani *et al.* 2007), and during a selective response only to relevant target objects (Hampshire *et al.* 2001). Mitchell *et al.* (2009) also demonstrated that the IFG was involved in resolving decision conflict during an instrumental learning task but only where a suboptimal response had been made; this might also be the case during the reversal stages following a shift in IED in the present study and fits with decreased volume in the inferior frontal cortex and poorer performance in patients. Previous research with alcoholic patients has shown that perseverative responses and perseverative errors in the WCST (an homologous task to IED) correlated negatively with superior temporal gyrus and postcentral gyrus volumes, an effect not replicated in the present study (Jang *et al.* 2010).

Social cognition as measured by emotional facial expression recognition was impaired in the alcoholic patients, albeit only fear recognition deficits were linearly related to detoxifications (fear recognition in controls > SDTx > MDTx). This finding supports previous findings (Kornreich *et al.* 2001; Townshend & Duka 2003). Interestingly, correct fear recognition was negatively associated with the volume of gray matter in IFG, an area involved in cognitive inhibitory control. It seems that perception of emotion from the fearful faces was not regulated to allow correct recognition probably because of a deficient IFG. It is of interest to note that in a *post hoc* analysis, we found a negative relationship between trait anxiety ratings and recognition of fearful faces (r = −0.358, *P *= 0.005). It is possible that increased anxiety enhances emotional input and arousal putting increasing demands to inhibitory control of emotion by IFG. It is worth noting that IFC may have some specific role in fearful expression recognition [e.g. Rahko *et al.* (2010) showed that in adolescents mirror neuron system in IFG activated more when viewing fearful compared with happy faces].

Recognition deficits for disgust and anger were only seen in the SDTx patients compared with controls. It is difficult to understand why this deficit was only seen in the SDTx patients.

In the VBM ANCOVA analysis, we used a conservative threshold of *P* < 0.05 FWE corrected. While this method protects against false positives, for modest effect sizes, the spatial extent of clusters may be reduced. Hence clusters in our analysis were typically small in spatial extent (one to eight voxels) although peak z scores were large (z > 4.8).

Disgust recognition was associated with gray matter volume in insula, parietal lobule and medial frontal gyrus. Insula is a central area for disgust recognition (e.g. Jehna *et al.* 2011). Relationship to parietal lobule and motor planning areas gray matter volume may be associated to some difficulty in recognizing disgust expressions; because of that, allocation of attention, supported by the inferior parietal lobule and planning of a motor response (Rushworth *et al.* 2005), supported by medial frontal gyrus are more in demand than during recognition of fearful expression.

Thus the present study has provided evidence that repeated episodes of detoxification from alcohol, related to the degree of alcohol dependency, are associated with altered brain structures and deficits in cognitive flexibility and recognition of emotional expressions in others. Such changes may confer increased inability to switch behavior according to environmental demands and increased social incompetence, contributing to relapse.
